# Symptomatic neurosyphilis in HIV-negative patients: a retrospective cohort study

**DOI:** 10.3389/fpubh.2025.1505818

**Published:** 2025-02-05

**Authors:** Qiaoer Chen, Weiqi Wu, Lu Wang, Honghong Huang, Lingxing Wang

**Affiliations:** ^1^Department of Neurology, Second Affiliated Hospital of Fujian Medical University, Quanzhou, Fujian, China; ^2^Department of Neurology, The Second Hospital of Longyan, Longyan, Fujian, China

**Keywords:** neurosyphilis, *Treponema pallidum*, HIV-negative individuals, interstitial neurosyphilis, parenchymal neurosyphilis

## Abstract

**Introduction:**

Neurosyphilis does not necessarily involve progressive invasion from interstitial to parenchymal nerve tissue. Few studies have focused on human immunodeficiency virus (HIV)-negative patients with symptomatic neurosyphilis, and the clinical outcomes and factors influencing the outcomes are unclear. Therefore, in this study, we aimed to compare the characteristics and clinical outcomes of interstitial and parenchymal neurosyphilis following treatment in HIV-negative patients with symptomatic neurosyphilis.

**Methods:**

We conducted a retrospective analysis of the clinical characteristics, laboratory results, neuroimaging findings, treatment regimens, and outcomes at discharge of HIV-negative patients admitted to our hospital with symptomatic neurosyphilis between May 2013 and May 2023.

**Results:**

Of the 142 patients, the mean age was 56.6 ± 11.4 years, with 111 (78.2%) being men. The predominant clinical manifestations included psychological disorders, cognitive decline, and cranial nerve disorders. Overall, 134 (94.4%) patients had elevated cerebrospinal fluid (CSF) cell counts, 113 (79.6%) had elevated protein levels, and 22/133 (16.5%) had elevated intracranial pressure. During hospitalization 113 patients (87.6%) were treated with intravenous penicillin and 13 (10.1%) were treated with ceftriaxone. Furthermore, 62 (43.7%) and 80 (56.3%) patients had interstitial and parenchymal types of neurosyphilis, respectively. Patients with the parenchymal type were younger and had higher platelet-to-lymphocyte ratio (PLR) and lower lymphocyte-to-monocyte ratio (LMR). Overall, 126 (88.7%) patients completed anti-syphilitic treatment prior to discharge, with 111 (88.1%) showing poor outcomes.

**Discussion:**

An elevated CSF protein level and the parenchymal type were associated with poor outcome. This study revealed that clinical manifestations of neurosyphilis vary, and that the majority of patients had elevated CSF cell and protein levels and a normal intracranial pressure. The PLR was higher and the LMR was lower in the parenchymal type than in the interstitial type. Only a small proportion of patients had favorable outcomes. CSF protein level and parenchymal type may be risk factors for poor prognosis.

## 1 Introduction

Syphilis is a chronic, systemic, sexually transmitted infection caused by *Treponema pallidum* that can affect any organ. Neurosyphilis, which occurs in 4–10% of syphilis cases (1), was historically viewed as a late-stage manifestation; however, recent research suggests that it can occur at any stage of infection (2). Neurosyphilis is classified into early and late stages based on the duration of infection, posing diagnostic challenges owing to the difficulty in confirming the time of infection onset. Therefore, clinical differentiation depends on the extent of tissue involvement and clinical presentation. *Treponema pallidum* predominantly targets the meningeal nerves and blood vessels, leading to interstitial neurosyphilis, which can manifest as meningitis, cranial nerve damage, polyradiculoneuropathy, and cerebrovascular disease (3). Parenchymal neurosyphilis is characterized by invasion of the cerebrospinal parenchyma, leading to general paresis and tabes dorsalis (3). Not all patients with neurosyphilis experience a spread from the interstitial tissue to the parenchymal tissue, and the invasion pattern of *Treponema pallidum* is unpredictable. Some studies suggest that CSF protein levels are higher in parenchymal neurosyphilis than in interstitial neurosyphilis (4); however, research on the distinctions between the two types is limited.

Most current research focuses on early non-invasive prediction of symptomatic neurosyphilis in asymptomatic patients (5, 6). However, in clinical practice, most patients present for medical evaluation after the onset of neurological manifestations, and the clinical manifestations and prognoses of symptomatic patients with neurosyphilis vary greatly. Moreover, the characteristics of symptomatic neurosyphilis in HIV-negative patients differ from those in patients with HIV infection. Patients with HIV infection have a higher incidence of neurosyphilis (7), and are more likely to have atypical presentation, false positive serological or CSF test results (8), and treatment failure (9) than HIV-negative patients with neurosyphilis. Few studies (10, 11) have been focused solely on HIV-negative patients with symptomatic neurosyphilis.

Patients with neurosyphilis have a low mortality rate; however, they, especially patients with parenchymal neurosyphilis, experience severe deterioration in mental and physical health, which significantly affects daily functioning (12). The clinical cure rate of neurosyphilis is 18–44% (13), but research on treatment outcomes is limited and the proportion of patients with favorable outcomes differs in different studies (14, 15). Moreover, the outcome on hospital discharge and factors influencing these outcomes in HIV-negative patients with symptomatic neurosyphilis at discharge are unclear. Therefore, in this study, we retrospectively reviewed the hospitalization data of patients with neurosyphilis treated at our hospital from 2013 to 2023. This study aimed to compare the characteristics of patients with interstitial and parenchymal neurosyphilis and evaluate the clinical outcomes at hospital discharge after treatment, along with the factors associated with a poor outcome, in HIV-negative patients with symptomatic neurosyphilis.

## 2 Materials and methods

### 2.1 Design and setting

This retrospective cohort study reviewed the general characteristics, clinical presentation, laboratory findings, and imaging features of HIV-negative patients with symptomatic neurosyphilis who were discharged from our hospital, a tertiary university hospital in Quanzhou, Fujian, China, between May 2013 and May 2023 using data from the electronic medical records.

### 2.2 Patient characteristics

The study cohort comprised individuals without HIV with a confirmed diagnosis of neurosyphilis and neurological manifestations. The diagnostic criteria for neurosyphilis adhered to the US Centers for Disease Control and Prevention *Sexually Transmitted Infections Treatment Guidelines* (7) or the Chinese Center for Disease Control and Prevention *Guidelines for* Diagnosis *and* Treatment *of* Syphilis*, Gonorrhea, and Genital Chlamydia trachomatis Infection* (16). The inclusion criteria were patients with: (1) positive results on blood non-treponemal and treponemal tests; (2) a CSF leukocyte count exceeding 5 × 10^6^ cells/L, or protein level greater than 500 mg/L, concomitant with a positive CSF fluorescent treponemal antibody absorption test, Venereal Disease Research Laboratory (VDRL) test, *Treponema pallidum* particle agglutination (TPPA) test, rapid plasma reagin (RPR), or toluidine red unheated serum test; and (3) presence of neurological manifestations. The exclusion criteria were patients with: (1) HIV coinfection, (2) incomplete clinical data, (3) comorbidities caused by other central nervous system infections, and (4) asymptomatic neurosyphilis. The Ethics Committee of the Second Affiliated Hospital of Fujian Medical University approved this study (No.: 2023–607). The requirement for informed consent was waived owing to its retrospective design.

### 2.3 Data collection

Clinical, laboratory, and imaging data were retrospectively extracted from the electronic medical records for analysis. The clinical data included demographic characteristics, such as age, sex, clinical symptoms and signs, and medical history, including hypertension, diabetes, and anti-syphilis treatment. Lifestyle factors such as smoking and alcohol consumption were also recorded. Laboratory investigations included blood and CSF tests. Imaging findings from computed tomography (CT) or magnetic resonance imaging (MRI) scans were also recorded. Complex inflammatory indices based on peripheral blood components, including the neutrophil-to-lymphocyte ratio (NLR), lymphocyte-to-monocyte ratio (LMR), platelet-to-monocyte (PMR), platelet-to-lymphocyte ratio (PLR), monocyte-to-high-density lipoprotein ratio (MHDLR), hemoglobin-to-lymphocyte ratio (HLR), hemoglobin-to-red blood cell distribution width ratio (HRR), and mean platelet volume-to-lymphocyte ratios (MPVLR), were calculated.

Based on the medical history, clinical manifestations, and clinical investigations, patients were categorized as having meningeal nerve syphilis, meningeal vascular syphilis, gummatous neurosyphilis, general paresis, or tabes dorsalis, as previously described (17). Meningeal and meningeal vascular syphilis, along with gummatous neurosyphilis, were classified as interstitial neurosyphilis, whereas general paresis and tabes dorsalis were categorized as parenchymal neurosyphilis (3).

### 2.4 Outcome assessment at discharge

The outcomes of patients who underwent complete anti-syphilis treatment during hospitalization were assessed on discharge using the Glasgow Outcome Scale (15). This scale ranges from 1 (death) to 5 (no or mild sequelae), with intermediate levels indicating varying degrees of severity: 2 (vegetative state), 3 (serious sequelae requiring daily care), and 4 (moderate sequelae with ability to live independently). A discharge Glasgow Outcome Scale score within the range of 1–4 was considered indicative of a poor outcome, whereas a score of 5 indicated a favorable outcome.

### 2.5 Statistical analysis

Statistical analyses were performed using SPSS version 27.0 (IBM Corp., Armonk, NY, USA). Descriptive statistics were used to summarize the data: continuous variables with a normal distribution were described using the mean ± standard deviation (SD), whereas those with non-normal distributions were described using median and interquartile range (IQR); and categorical variables were described using the frequency and percentage. Groups were compared using t-tests for normally distributed data, non-parametric tests of non-normally distributed data, and chi-square tests for categorical data. Binary logistic regression was used to assess the factors associated with clinical outcomes at discharge following anti-syphilitic treatment. Statistical significance was set at *p* < 0.05 for all analyses.

## 3 Results

### 3.1 Demographic and clinical characteristics

**Figure 1:**
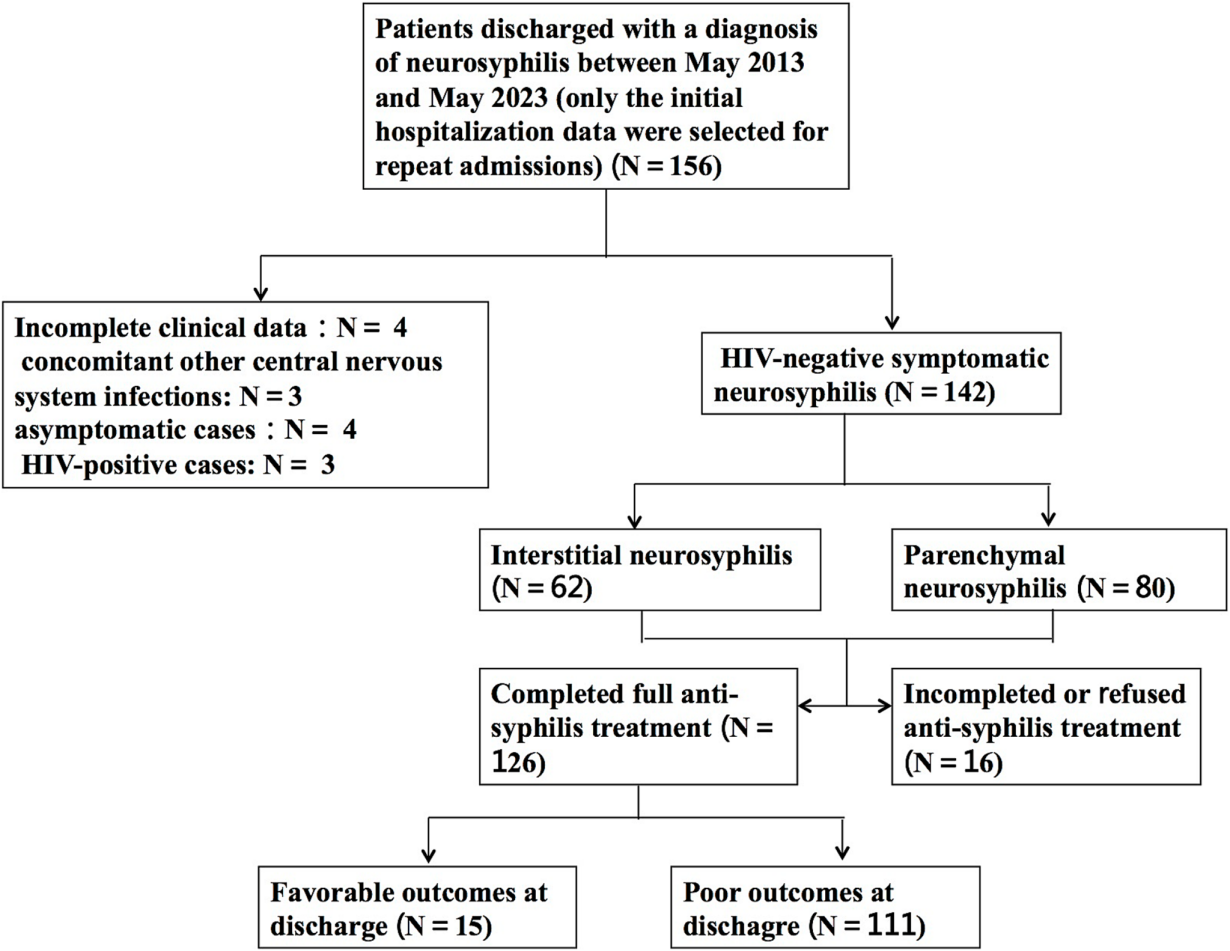
Flow chart for selection of HIV-negative patients with symptomatic neurosyphilis.

In total, 142 HIV-negative patients diagnosed with symptomatic neurosyphilis were included based on the predefined inclusion and exclusion criteria. Figure 1 shows a study flowchart of patient selection. All 142 eligible patients had complete clinic data and blood test results, including complete blood count, biochemical tests, blood TPPA, and blood RPR, as well as cerebrospinal fluid (CSF) examination results. Additionally, 106 patients underwent brain MRI. The patients had a mean age of 56.6 ± 11.4 years, with 54 (38%) aged between 51 and 60 years, 34 (24%) aged 61–70 years, and 29 (20%) aged 41–50 years. The cohort comprised 111 (78.2%) men and 31 (21.8%) women. Among them, 52 (36.6%) had hypertension, 19 (13.4%) had diabetes, 34 (23.9%) had a history of smoking, 23 (16.2%) had a history of alcohol consumption, and 12 (8.5%) were diagnosed with syphilis before admission. The most prevalent clinical manifestations among the 142 patients were psychological disorders, including mood changes, delusions, mania, and confusion in 43 patients (30.3%); cognitive decline in 42 patients (29.6%); and cranial nerve disorders in 31 patients (21.8%), of whom 13 (9.2%) presented with blurred vision. Ophthalmological examinations revealed optic atrophy in 2 patients and bilateral uveitis in 1 patient. Moreover, speech impairment, hemiplegia, sensory disturbance, disturbance of consciousness, and Argyll-Robertson pupil were found in 27 (19.0%), 21 (14.8%), 14 (9.9%), 9 (6.3%), and 6 (4.2%) patients, respectively (Table 1).

**Table 1:** Clinical, blood, cerebrospinal fluid and MRI findings of patients with HIV-negative symptomatic neurosyphilis.

Variables	n/N (%)
Clinical manifestations	
Psychological disorder	43/142 (30.3)
Cognitive decline	42/142 (29.6)
Cranial nerve disorder	31/142 (21.8)
Speech impairment	27/142 (19.0)
Dizziness	27/142 (19.0)
Hemiplegia	21/142 (14.8)
Sensory disturbance	14/142 (9.9)
Epilepsy	12/142 (8.5)
Headache	11/142 (7.7)
Disturbance of consciousness	9/142 (6.3)
Nausea or vomiting	6/142 (4.2)
Argyll-Robertson pupil	6/142 (4.2)
Blood test results	
Elevated blood WBC (>9.5 × 10^9^/L)	30/142 (21.1)
Positive blood TPPA	142/142 (100)
Positive blood RPR	142/142 (100)
Intracranial pressure (mmH_2_O)	
Normal (80–180 mmH_2_O)	107/133 (80.5)
Elevated (≥180 mmH_2_O)	22/133 (16.5)
Reduced (<80 mmH_2_O)	4/133 (3.0)
CSF tests results	
Elevated CSF WBC (≥5 × 10^6^/L)	134/142 (94.4)
Reduced CSF glucose (<2.5 mmol/L)	9/142 (6.3)
Elevated CSF protein (>50 mg/dL)	113/142 (79.6)
Positive CSF TPPA	135/135 (100)
Positive CSF RPR	120/142 (84.5)
Brain MRI	
Cerebral atrophy	40/106 (37.7)
Periventricular hyper-intense signal	28/106 (26.4)
Cerebral infarction	24/106 (22.6)
Lacunar focus	8/106 (7.5)
Intracranial mass (gummatous neurosyphilis)	3/106 (2.8)
Cerebral hemorrhage	3/106 (2.8)
Encephalitis	3/106 (2.8)
Hydrocephalus	2/106 (1.9)

CSF, cerebrospinal fluid; MRI, magnetic resonance imaging; RPR, rapid plasma regain test; TPPA, Treponemal pallidum particle agglutination test; WBC, white blood cell.

### 3.2 Blood, CSF, and imaging results

Among the 142 patients, 30 (21.1%) had elevated white blood cell counts in the blood. All patients had positive blood TPPA and RPR test results (Table 1). Lumbar puncture was performed on all patients on admission, with 133 (93.7%) undergoing intracranial pressure (ICP) measurement, of whom 107 (80.5%) had normal intracranial pressure (80–180 mmH_2_O), 22 (16.5%) had elevated pressure, and 4 (3.0%) had decreased pressure. Additionally, 134 patients (94.4%) had increased CSF cell counts, 9 (6.3%) had decreased CSF glucose levels, and 113 (79.6%) had elevated CSF protein levels. The CSF TPPA test was performed in 135 patients (95.1%) and all were positive, whereas CSF RPR test was positive in 120 patients (84.5%) and negative in 22 patients (15.5%) (Table 1).

All 142 patients underwent brain MRI, or CT, or both. Of the patients, 106 (74.6%) underwent brain MRI and 16 (11.3%) underwent spinal MRI. Among the 106 patients who underwent brain MRI, cerebral atrophy was observed in 40 (37.7%), periventricular hypersignals in 28 (26.4%), infarction in 24 (22.6%), and lacunar lesions in 8 (7.5%). Additionally, three (2.8%) patients had an intracranial mass (subsequently confirmed as gummatous neurosyphilis on postoperative pathology), three (2.8%) had cerebral hemorrhage, three (2.8%) had signs of encephalitis, and two (1.9%) had signs of hydrocephalus (Table 1). Among the 16 patients who underwent spinal MRI, four (25%) had abnormal signals detected.

### 3.3 Treatment

Among the 142 patients, 13 (9.2%) declined anti-syphilis treatment, 126 (88.7%) were treated with antibiotics, and 3 (2.1%) underwent surgery. The three patients who underwent surgery were confirmed to have neurosyphilis on postoperative pathology and subsequently received antibiotic treatment. Among the 129 patients who received antibiotic treatment after admission, 113 (87.6%) received intravenous penicillin at a dose of 18–24 million U/day for 10–14 days, followed by three doses of benzathine penicillin 2.4 million U/week via intramuscular injection, with the second and third doses provided as outpatient treatment. Thirteen (10.1%) patients received ceftriaxone (2 g/day) for 10–14 days, and three (2.3%) patients received oral doxycycline 100 mg twice daily for 30 days.

### 3.4 Comparison of the characteristics of the interstitial and parenchymal types

Among the 142 patients, 62 (43.7%) had the interstitial type, including 24 (38.7%) with meningeal involvement, 35 (56.5%) with meningovascular complications, and 3 (4.8%) with gummatous lesions. The remaining 80 (56.3%) patients had the parenchymal type, of whom 66 (82.5%) had general paresis and 14 (17.5%) had tabes dorsalis. Patients with the interstitial type were significantly older, with significantly higher rates of hypertension and diabetes, and a significantly shorter symptom duration than those with parenchymal involvement (Table 2); however, sex, smoking history, and alcohol consumption did not differ significantly between the two groups. Cranial nerve disorders, hemiplegia, speech impairment, and dizziness were significantly more common clinical manifestations in the interstitial type, whereas psychological disorders and cognitive decline were significantly more common in the parenchymal type, with no significant differences in the other clinical manifestations between the two types.

**Table 2:** Comparison of the characteristics of interstitial and parenchymal types of neurosyphilis in HIV-negative patients.

	Interstitial (N = 62)	Parenchymal (N = 80)	*P*
Age (years), mean ± SD	60.1 ± 11.9	53.9 ± 10.3	0.001*
Male, *n* (%)	49 (79.0)	62 (77.5)	0.826
Hypertension, *n* (%)	31 (50.0)	21 (26.3)	0.004*
Diabetes, *n* (%)	13 (21.0)	6 (7.5)	0.019*
Smoking, *n* (%)	16 (25.8)	18 (22.5)	0.647
Alcohol consumption, *n* (%)	9 (14.5)	14 (17.5)	0.632
Symptom duration (days), median (IQR)	6.9 (0.9–30.0)	180 (9.9–360)	<0.001*
Psychological disorder, *n* (%)	1 (1.6)	42 (52.5)	<0.001*
Cognitive decline, *n* (%)	3 (4.8)	39 (48.8)	<0.001*
Cranial nerve disorder, *n* (%)	27 (43.5%)	4 (5.0)	<0.001*
Speech impairment, *n* (%)	18 (29.0)	9 (11.3)	0.007*
Dizziness, *n* (%)	18 (29.0)	9 (11.3)	0.007*
Hemiplegia, *n* (%)	21 (33.9)	0 (0)	<0.001*
Sensory disturbance, *n* (%)	6 (9.7)	8 (10.0)	0.949
Epilepsy, *n* (%)	5 (8.1)	7 (8.8)	0.884
Headache, *n* (%)	5 (8.1)	6 (7.5)	>0.999
Disturbance of consciousness, *n* (%)	7 (11.3)	2 (2.5)	0.074
Nausea or vomiting, *n* (%)	4 (6.5)	2 (2.5)	0.459

*p < 0.05; IQR, interquartile range; SD, standard deviation.

High-density lipoprotein levels were significantly higher in the parenchymal type than in the interstitial type, but no significant differences were observed in other blood parameters, including the RPR titer, white blood cell count, or cholesterol, low-density lipoprotein, and blood homocysteine levels. Similarly, no significant differences were observed in CSF indices between the two types (Table 3). Patients with parenchymal neurosyphilis had a significantly lower LMR and a significantly higher PLR than those with interstitial neurosyphilis. However, no significant differences in the other composite inflammatory indicators including NLR, PMR, MHDLR, HLR, HRR, and MPVLR, were observed between the two types.

**Table 3:** Blood, cerebrospinal fluid and MRI comparisons between interstitial and parenchymal types in HIV-negative symptomatic patients.

	Interstitial (*N* = 62)	Parenchymal (*N* = 80)	*p*
CSF tests			
CSF WBC (×10^6^/L), median (IQR)	34.00 (10.00–85.25)	28.00 (8.00–56.75)	0.110
CSF protein (mg/dL), median (IQR)	74.65 (57.55–101.15)	77.00 (52.00–109.59)	0.979
CSF glucose (mmol/L), median (IQR)	3.36 (3.05–4.14)	3.37 (2.99–3.84)	0.422
CSF RPR titer, median (IQR)	2 (1–8)	4 (1.25–8)	0.368
Blood tests			
Blood RPR titer, median (IQR)	32 (8–80)	32 (16–64)	0.915
WBC (×10^9^/L), median (IQR)	7.52 (6.05–9.05)	7.50 (6.31–9.27)	0.547
Blood neutrophils (×10^9^/L), median (IQR)	4.85 (3.70–6.38)	5.20 (3.93–6.95)	0.270
Blood lymphocytes (×10^9^/L), mean ± SD	1.82 ± 0.69	1.64 ± 0.67	0.132
Blood monocytes (×10^9^/L), median (IQR)	0.50 (0.32–0.71)	0.56 (0.40–0.61)	0.482
Red blood cells (×10^12^/L), median (IQR)	4.59 (4.27–4.81)	4.54 (4.20–4.86)	0.894
Blood platelets (×10^9^/L), median (IQR)	246 (201.5–283.25)	257 (225–290.5)	0.149
Hemoglobin (g/L), median (IQR)	135 (129–145)	135 (128–145.25)	0.666
Platelet volume (fL), mean ± SD	9.70 ± 0.77	9.65 ± 0.91	0.749
Cholesterol (mmol/L), mean ± SD	4.47 ± 1.08	4.76 ± 0.95	0.094
HDL (mmol/L), median (IQR)	1.11 (0.87–1.32)	1.22 (1.02–1.60)	0.017*
LDL (mmol/L), mean ± SD	2.70 ± 0.89	2.84 ± 0.82	0.326
Total bilirubin (μmol/L), median (IQR)	10.01 (7.34–13.50)	8.94 (6.75–11.85)	0.129
Blood homocysteine (μmol/L), median (IQR)	12.79 (10.60–14.15)	14.34 (10.99–14.52)	0.079
NLR, median (IQR)	2.67 (1.95–3.84)	3.26 (2.36–5.00)	0.055
LMR, median (IQR)	3.71 (2.85–4.85)	3.11 (2.23–4.11)	0.010*
PLR, median (IQR)	132.26 (109.69–177.56)	159.69 (127.50–217.26)	0.010*
PMR, median (IQR)	532.10 (351.48–743.75)	467.98 (397.86–620.21)	0.796
MHDLR, median (IQR)	0.42 (0.29–0.67)	0.45 (0.27–0.58)	0.448
HLR, median (IQR)	80.58 (62.52–102.06)	84.09 (65.05–105.29)	0.320
HRR, median (IQR)	10.37 (9.50–11.33)	10.35 (9.52–1.20)	0.660
MPVLR, median (IQR)	5.63 (4.32–7.89)	6.28 (4.54–7.71)	0.194
Brain MRI, n/N (%)			
Cerebral atrophy	13/51 (25.5)	27/55 (49.1)	0.012^*^
Periventricular hyper-intense signal	14/51 (27.5)	14/55 (25.5)	0.816
Cerebral infarction	24/51 (47.1)	0/55 (0)	<0.001^*^
Lacunar focus	4/51 (7.8)	4/55 (7.3)	>0.999
**Antibiotic treatment, *n* (%)**			0.736
Penicillin	48 (77.4)	65 (81.3)	
Ceftriaxone	7 (11.3)	6 (7.5)	
Incomplete	7 (11.3)	9 (11.3)	

HDL, high-density lipoprotein; HLR, hemoglobin-to-lymphocyte ratio; HRR, hemoglobin-to-red blood cell distribution width ratio; LDL, low-density lipoprotein; LMR, lymphocyte-to-monocyte ratio; MHDLR, monocyte-to-high-density lipoprotein ratio; MPVLR, mean platelet volume-to-lymphocyte ratio; NLR, neutrophil-to-lymphocyte ratio; PMR, platelet-to-monocyte ratio; PLR, platelet-to-lymphocyte ratio. *p < 0.05.

Of the 142 patients, 106 (74.6%) underwent brain MRI, of whom 51 (48.1%) had the interstitial type, and 55 (51.9%) had the parenchymal type. Cerebral infarction was significantly more common in the interstitial type, whereas cerebral atrophy was significantly more common in the parenchymal type (Table 3). No significant differences in periventricular hyperintensities or lacunar foci were observed between the two types.

The antibiotic treatment did not differ significantly between the two types. Among patients with the interstitial type, 48 (77.4%) received penicillin, 7 (11.3%) received ceftriaxone, and 7 (11.3%) did not complete antibiotic treatment. Among patients with the parenchymal type, 65 (81.3%) received penicillin, 6 (7.5%) received ceftriaxone, and nine (11.3%) did not complete antibiotic treatment.

### 3.5 Factors associated with clinical outcomes at discharge

Among the 142 patients, 126 (88.7%) completed full anti-syphilitic treatment on discharge, with a mean antibiotic duration of 12.05 ± 0.97 days. At discharge, 15 (11.9%) patients had favorable outcomes based on a Glasgow Outcome Scale score of 5, whereas 111 (88.1%) showed poor outcomes. Favorable outcomes were significantly more common in patients with the interstitial type than in those with the parenchymal type (21.8% vs. 4.2%) (Table 4). Sex, age, hypertension, diabetes, smoking history, and alcohol consumption, prior anti-syphilis treatment, and symptom duration at diagnosis did not differ significantly between the two groups. Comparatively, the poor outcome group had significantly higher CSF protein levels, whereas no significant differences were observed in CSF cell count, glucose, chloride, or RPR titers. Patients with favorable outcomes had significantly higher white blood cell and lymphocyte counts, whereas the blood RPR titer, neutrophil count, monocyte count, neutrophil ratio, lymphocyte ratio, monocyte ratio, and various blood parameters did not differ significantly between the favorable and poor outcome groups. Furthermore, the HLR and MPVLR were significantly lower in the favorable outcome group. However, no significant differences in the NLR, LMR, PLR, PMR, MHDLR, and HRR were observed between groups. The outcome did not differ significantly according to the type of antibiotic treatment. Logistic regression analysis revealed that CSF protein levels and neurosyphilis type were significantly associated with the clinical outcomes at discharge (Table 4).

**Table 4:** Risk factors for clinical outcomes of HIV-negative symptomatic neurosyphilis patients at discharge.

	Favorable outcome (*n* = 15)	Poor outcome (*n* = 111)	*p*	OR (95% CI)	*p*
Sex, *n* (%)			>0.999		
Male	11 (73.3)	84 (75.7)			
Female	4 (26.7)	27 (24.3)			
Age (years), mean (SD)	55.6 ± 12.0	57.3 ± 11.0	0.577		
Hypertension, *n* (%)	5 (33.3)	43 (38.7)	0.686		
Diabetes, *n* (%)	1 (6.7)	16 (14.4)	0.673		
Smoking, *n* (%)	3 (20.0)	25 (22.5)	>0.999		
Alcohol consumption, *n* (%)	1 (6.7)	18 (16.2)	0.558		
Prior anti-syphilis treatment, *n* (%)	4 (26.7)	8 (7.2)	0.052		
Symptom duration (days), median (IQR)	30 (2.1–120)	30 (3–180)	0.583		
Type, *n* (%)			0.002^*^	6.69 (1.40–31.9)	0.017^*^
Interstitial	12 (80)	43 (38.7)			
Parenchymal	3 (20)	68 (61.3)			
CSF WBC (10^6^/L), median (IQR)	27 (10, 80)	31 (8, 69.5)	0.590		
CSF Protein (mg/dL), median (IQR)	55.0 (47.4–77.6)	80.4 (60.0–109.95)	0.044^*^	1.03 (1.00–1.05)	0.049^*^
CSF Glu (mmol/L), median (IQR)	3.30 (3.11–3.50)	3.37 (2.96–3.99)	0.985		
CSF RPR titer, median (IQR)	4 (2–8)	4 (2–8)	0.816		
Blood RPR titer, median (IQR)	16 (8–64)	32 (16–64)	0.349		
WBC (×10^9^/L), median (IQR)	8.40 (7.58–10.40)	7.20 (6.00–8.94)	0.010^*^	3.80 (0.34–42.3)	0.278
Blood neutrophils (×10^9^/L), median (IQR)	6.10 (5.00–7.20)	4.73 (3.80–6.30)	0.059	0.16 (0.01–2.13)	0.167
Blood lymphocytes (×10^9^/L), mean ± SD	1.99 ± 0.69	1.60 ± 0.61	0.025^*^	1.09 (0.04–33.7)	0.960
Blood monocytes (×10^9^/L), median (IQR)	0.59 (0.42–0.80)	0.50 (0.32–0.60)	0.128		
Red blood cells (×10^12^/L), median (IQR)	4.77 (4.23–4.89)	4.51 (4.20–4.80)	0.335		
Blood platelets (×10^9^/L), median (IQR)	256 (208–287)	252 (204–284)	0.598		
Hemoglobin (g/L), median (IQR)	135 (129–145)	135 (128–144)	0.772		
Platelet volume (fL), mean ± SD	9.70 ± 0.86	9.67 ± 0.85	0.903		
Cholesterol (mmol/L), mean ± SD	4.74 ± 1.29	4.63 ± 0.94	0.687		
HDL (mmol/L), median (IQR)	1.22 (1.08–1.52)	1.20 (0.94–1.48)	0.362		
LDL (mmol/L), mean ± SD	2.76 ± 0.93	2.77 ± 0.79	0.950		
Total bilirubin (μmol/L), median (IQR)	10.85 (6.58–14.50)	9.19 (7.20–12.30)	0.460		
Blood homocysteine (μmol/L), median (IQR)	12.72 (11.34–13.31)	13.39 (10.63–14.72)	0.271		
NLR, median (IQR)	2.89 (2.32–4.24)	3.17 (2.07–4.83)	0.934		
LMR, median (IQR)	3.67 (2.17–4.67)	3.25 (2.40–4.50)	0.593		
PLR, median (IQR)	138.67 (109.55–157.06)	155.19 (120.34–212.64)	0.091		
PMR, median (IQR)	473.33 (352.50–600.00)	484.00 (406.00–737.50)	0.498		
MHDLR, median (IQR)	0.41 (0.26–0.58)	0.41 (0.27–0.59)	0.635		
HLR, median (IQR)	69.47 (53.31–90.00)	86.25 (67.89–111.28)	0.041^*^	1.01 (0.94–1.09)	0.718
HRR, median (IQR)	10.71 (9.28–12.14)	10.35 (9.52–11.14)	0.436		
MPVLR, median (IQR)	4.77 (4.13–6.15)	6.33 (4.81–8.42)	0.045^*^	1.73 (0.55–5.39)	0.348
Treatment			>0.999		
Penicillin, *n* (%)	13 (86.7)	100 (90.1)			
Ceftriaxone, *n* (%)	2 (13.3)	11 (9.9)			

HDL, high-density lipoprotein; HLR, hemoglobin-to-lymphocyte ratio; HRR, hemoglobin-to-red blood cell distribution width ratio; LDL, low-density lipoprotein; LMR, lymphocyte-to-monocyte ratio; MHDLR, monocyte-to-high-density lipoprotein ratio; MPVLR, mean platelet volume-to-lymphocyte ratio; NLR, neutrophil-to-lymphocyte ratio; PMR, platelet-to-monocyte ratio; PLR, platelet-to-lymphocyte ratio. *p < 0.05.

## 4 Discussion

This study included 142 HIV-negative patients with symptomatic neurosyphilis based on an electronic medical record review of patients admitted to our hospital over the past decade. The majority of patients were male, with a mean age of 56.6 years, peaking in the 51–60-year age group, consistent with previous studies (15, 18). Interstitial neurosyphilis typically occurs several months to 10 years after syphilis infection, whereas parenchymal neurosyphilis typically manifests 10–30 years after infection. Therefore, patients diagnosed with parenchymal neurosyphilis were expected to have a higher average age at onset than those with interstitial neurosyphilis. Unexpectedly, this study revealed that the age at hospital admission was lower in patients with parenchymal neurosyphilis than in those with interstitial neurosyphilis. There is limited data specifically addressing age differences between parenchymal and interstitial neurosyphilis in HIV-negative patients and further studies are needed to clarify this observation. Moreover, *Treponema pallidum*, known as the “great mimic,” can simulate various diseases, and neurosyphilis can present with complex and atypical clinical manifestations, which can delay the diagnosis. Ozturk-Engin et al. (19) identified headache as the predominant symptom in patients with neurosyphilis. Several studies (20–22) have highlighted dementia as the primary manifestation. Consistent with these results, psychiatric manifestations were the most frequent in this study.

In this study, only 19.5% of patients who underwent CSF pressure measurement had abnormal pressure, whereas 94.4% had an increased cell count and 79.6% had an elevated CSF protein level. These results suggest that HIV-negative individuals may be more prone to changes in CSF cell counts and protein levels, with less impact on pressure. Moreover, no significant differences were observed in the CSF parameters between the interstitial and parenchymal types. Owing to the invasive nature of lumbar puncture, the relationship between peripheral blood inflammatory composite indicators and neurosyphilis has attracted attention. Previous studies (23) have reported that NLR levels are higher in patients with neurosyphilis compared to those with syphilis without neurological involvement; however, the differential expression of composite inflammatory indicators according to the neurosyphilis type has not been reported previously. This study found no difference in NLR expression between patients with the interstitial and parenchymal types of neurosyphilis; however, the LMR was significantly lower, and the PLR was significantly higher, in patients with parenchymal neurosyphilis than in those with interstitial neurosyphilis. Monocytes and platelets release proinflammatory cytokines in response to infection, and subsequent adaptive responses by lymphocytes balance these cytokines, resulting in an effective immune response that prevents excessive inflammation while clearing the infection (23–25). A lower LMR may reflect a higher degree of monocyte activation, which is known to play a role in chronic inflammation and tissue destruction (26). Monocytes and macrophages can infiltrate the central nervous system and contribute to the pathophysiology of neuroinflammation by producing pro-inflammatory cytokines, such as TNF-*α* and IL-6 (27). The more pronounced infiltration of monocytes in parenchymal neurosyphilis may explain the reduced LMR. In contrast, the higher PLR could indicate a more intense systemic inflammatory response. Platelets are not only involved in coagulation but also play a critical role in inflammation by interacting with immune cells, including monocytes and lymphocytes, and releasing inflammatory mediators (28). An elevated PLR suggests heightened platelet activation, which has been associated with various neurological disorders characterized by neuroinflammation (29). Multiple studies have indicated the utility of LMR and PLR in assessing inflammation in tuberculosis and cardiovascular diseases (30–32), and their diagnostic and prognostic value in osteoarthritis, malignant tumors, and Parkinson’s disease (33–37). Therefore, we hypothesized that decreased LMR and increased PLR could be linked to the nature of parenchymal neurosyphilis, which may involve longer disease duration or more advanced stages of infection, leading to greater immune dysregulation and chronic inflammatory processes (6) and even potentially offering insights into prognosis.

Current treatment antibiotic options for neurosyphilis include penicillin, ceftriaxone, or doxycycline. In this study, all HIV-negative patients with symptomatic neurosyphilis showed some degree of clinical improvement after antibiotic treatment, consistent with the findings of a previous study (14). However, only 11.9% had favorable outcomes at discharge, which is lower than that previously reported (14, 15). Variations in the inclusion criteria, HIV status, neurosyphilis type distribution, and follow-up duration may explain this discrepancy. Ozturk-Engin et al. (19) included asymptomatic and symptomatic cases and found neurological sequelae in 41.8% of patients. Excluding patients with parenchymal neurosyphilis, Bettuzzi et al. (14) reported a 37.0% good outcome rate after 1 month of treatment. In a study of HIV-positive and HIV-negative patients with neurosyphilis, Schnohr et al. (15) noted poor outcomes in 21% of patients at discharge, 19% at 1 month and 13% at 3 months post-discharge, with better outcomes in HIV-positive patients than in HIV-negative patients. This study focused solely on HIV-negative patients with symptomatic neurosyphilis, which might have contributed to a higher proportion of poor discharge outcomes. Retrospective studies cannot determine long-term outcomes; therefore, prospective studies with longer-term follow-up are warranted. Moreover, the findings revealed that CSF protein levels and neurosyphilis classification were independently associated with discharge outcomes. To our knowledge, this is the first study exploring factors affecting discharge outcomes restricted to HIV-negative individuals with symptomatic neurosyphilis. In contrast to the results of this study, Schnohr et al. (15) found that a CSF white blood cell count ≥30 × 10^6^/L was a predictor of poor outcomes after discharge in a study of neurosyphilis that included both symptomatic and asymptomatic patients. It has been reported (5) that a higher CSF protein content may indicate neurosyphilis progression and more severe neurological manifestations, which could explain our findings. The neurosyphilis type, particularly the parenchymal type, can also affect the effectiveness of treatment, often leading to cognitive impairment and psychiatric manifestations, resulting in suboptimal outcomes.

This study has some limitations. First, it was a retrospective, single-center study, which might limit the generalizability of the findings. Second, as the analysis relied on inpatient medical record data, we were unable to conduct post-discharge patient follow-up or assess dynamic changes in the blood serology and CSF results and were therefore unable to conduct a comprehensive evaluation of clinical treatment outcomes. These limitations highlight the need for multicenter prospective cohort studies. Another limitation is the lack of control for patients’ baseline health, including comorbidities, which may have affected treatment response and prognosis. Future studies should address these factors to better assess treatment outcomes in neurosyphilis.

## 5 Conclusion

In this cohort of HIV-negative patients with symptomatic neurosyphilis, the mean age was 56.6 years, with a male predominance. Psychiatric conditions were the most common clinical manifestation. Meningovascular syphilis and general paresis were the major types of interstitial and parenchymal neurosyphilis, respectively. Patients with the parenchymal type were younger and had higher PLR and lower LMR than those with the interstitial type. All patients showed some degree of improvement in the clinical manifestations after anti-syphilis treatment; however, only a small minority had favorable outcomes at discharge. The cerebrospinal fluid protein level and neurosyphilis type were independently associated with the discharge outcome.

## Data Availability

The raw data supporting the conclusions of this article will be made available by the authors, without undue reservation.
